# Knockdown of *hspg2* is associated with abnormal mandibular joint formation and neural crest cell dysfunction in zebrafish

**DOI:** 10.1186/s12861-021-00238-4

**Published:** 2021-03-08

**Authors:** Barbara S. Castellanos, Nayeli G. Reyes-Nava, Anita M. Quintana

**Affiliations:** Department of Biological Sciences, University of Texas El Paso, El Paso, TX 79968 USA

**Keywords:** *HSPG2*, Neural crest cells, Craniofacial development

## Abstract

**Background:**

Heparan sulfate proteoglycan 2 (*HSPG2)* encodes for perlecan, a large proteoglycan that plays an important role in cartilage formation, cell adhesion, and basement membrane stability. Mutations in *HSPG2* have been associated with Schwartz-Jampel Syndrome (SJS) and Dyssegmental Dysplasia Silverman-Handmaker Type (DDSH), two disorders characterized by skeletal abnormalities. These data indicate a function for *HSPG2* in cartilage development/maintenance. However, the mechanisms in which *HSPG2* regulates cartilage development are not completely understood. Here, we explored the relationship between this gene and craniofacial development through morpholino-mediated knockdown of *hspg2* using zebrafish.

**Results:**

Knockdown of *hspg2* resulted in abnormal development of the mandibular jaw joint at 5 days post fertilization (DPF). We surmised that defects in mandible development were a consequence of neural crest cell (NCC) dysfunction, as these multipotent progenitors produce the cartilage of the head. Early NCC development was normal in morphant animals as measured by distal-less homeobox 2a (*dlx2a)* and SRY-box transcription factor 10 (*sox10)* expression at 1 DPF. However, subsequent analysis at later stages of development (4 DPF) revealed a decrease in the number of Sox10 ^+^ and Collagen, type II, alpha 1a (Col2a1a)^+^ cells within the mandibular jaw joint region of morphants relative to random control injected embryos. Concurrently, morphants showed a decreased expression of *nkx3.2,* a marker of jaw joint formation, at 4 DPF.

**Conclusions:**

Collectively, these data suggest a complex role for *hspg2* in jaw joint formation and late stage NCC differentiation.

## Background

Mutation of the *HSPG2* gene causes Schwartz-Jampel Syndrome (SJS) and Dyssegmental Dysplasia Silverman-Handmaker Type (DDSH) [1–3]. SJS is a recessive disorder characterized by muscle stiffness (myotonia) and chondrodysplasia. DDSH is a less frequent but more severe recessive disorder characterized by reduced joint mobility, severe limb shortening, and short stature [4]. Both disorders have common clinical manifestations that include reduced stature, bowing of the long bones, and facial dimorphism [4, 5]. Interestingly, SJS is generally associated with loss of function mutations in *HSPG2* that vary from missense mutations to splice site mutations. These mutations are dispersed throughout the *HSPG2* protein, with those located closer to the C -terminus being more readily tolerated [5]. Conversely, DDSH is caused by homozygous null mutations (frameshifts and point mutations) wherein there is an absence of functional protein, likely due to degradation [5]. Despite mutation heterogeneity underlying SJS and DDSH, craniofacial abnormalities such as micrognathia are very common phenotypes in diagnosed patients [5]. These manifestations of chondrodysplasia suggest that *HSPG2* plays a role in regulating craniofacial development.

The function of *HSPG2* during chondrogenesis is not completely understood, but is likely related to the various domains within the *HSPG2* protein and the vast array of interactions between *HSPG2* and the extracellular matrix (ECM) to promote signal transduction and stability [6, 7]. *HSPG2* encodes for perlecan, a large proteoglycan that consists of five domains, each of which have a unique function [8]. Four of these domains (domains II, III, IV, and V) have repeats homologous to low-density lipoprotein receptor (domain II), laminins (domains III and V), and immunoglobulins (domain IV), but domain I is unique to perlecan [8]. The N-terminal domain I possesses three ser-asp-gly motifs that serve as attachment sites for glycosaminoglycan (GAG) side chains like heparan sulfate (HS) and chondroitin sulfate (CS) [8–10]. These side chains facilitate interaction with growth factors such as fibroblast growth factor-2 (FGF-2), vascular endothelial growth factors (VEGFs), and bone morphogenetic proteins (BMPs) [6, 10, 11] upon secretion of perlecan into the ECM. In addition, HS has been shown to specifically interact with ECM proteins such as fibronectin, laminin, and collagens I, II, III, IV, and V, suggesting perlecan and its side chains mediate cell adhesion and basement membrane stability [12].

As it relates to the chondrocyte phenotypes in SJS and DDSH, previous studies, both in vitro and in vivo, have established that disrupting *HSPG2* expression leads to abnormal chondrocyte proliferation and disorganized columnar arrangement [13, 14]. This is significant as chondrocytes are the primary cells found in cartilage and are known to secrete a specialized ECM containing glycoproteins and proteoglycans, like perlecan, in order to maintain structural integrity [15]. During craniofacial development, chondrocytes arise from neural crest cells (NCCs), a multipotent progenitor cell population that forms at the dorsal end of the neural tube upon neural tube closure. There are four populations of NCCs, but only cranial NCCs (CNCCs) migrate to the pharyngeal arches and develop into cartilage and bone, making them vital to proper craniofacial development [16–18]. These CNCCs are regulated by growth factors like BMP, which are vital for mandibular morphogenesis, and FGFs, which are essential for CNCC differentiation [19–21].

The development of craniofacial bone is due to both endochondral and intramembranous ossification. In endochondral ossification, cells differentiate into chondrocytes and bones develop from a cartilaginous base. This form of ossification necessitates that chondrocytes are present in growth plates where they are arranged into zones of rest, proliferation, and hypertrophy [15, 22]. These chondrogenic cells play a part in the development of the mandibular jaw joint (also known as the temporomandibular joint or TMJ in mammals) and are derived in part by CNCCs [23]. While the TMJ is a synovial joint comprised of the mandibular condyle and glenoid fossa, it develops slightly different than other synovial joints [23]. To form the TMJ, CNCCs migrate to the mandibular arch and develop into Meckel’s cartilage, which extends out of the mandibular process into the tympanic process. Therefore, middle ear development is tightly intertwined with TMJ development. As with other bone and joint development, the Meckel’s cartilage is eventually replaced by intramembraneous bone formation. During this process, a blastema, which forms the condylar secondary cartilage (CSC) develops. The CSC is located beneath a thin layer of periosteum [23]. The condyle is formed from the secondary cartilage and the periosteal space. It is made of fibrocartilage, which has a denser ECM than fibrous connective tissue. Ultimately, the fibrocartilage will become an articular disc that divides the fluid filled joint cavity into upper and lower compartments lined by a soft tissue known as synovium [23]. Thus, unlike other synovial joints, the TMJ is formed from CNCCs and the periosteum. In addition, it contains fibrocartilage, which is located in fewer regions/joints in the body and is distinct because it has a denser ECM than other fibrous tissue. Given the role of ECM and the periosteum in TMJ development, we hypothesized that deletion of perlecan, a primary component of the ECM, would disrupt jaw joint formation.

To begin to test this hypothesis, we performed morpholino mediated knockdown of *hspg2* using the zebrafish model. Apart from general advantages like a high fecundity rate, extra-uterine development, and quick maturation, the zebrafish is an ideal organism to study craniofacial development. This is due to the relative simplicity of the cartilaginous structures of the head and face, all of which are readily visible with stains like Alcian blue [24, 25]. Most importantly, CNCC development is highly conserved in zebrafish and consequently, suggests that zebrafish are an appropriate model to characterize the function of *hspg2* in craniofacial development [25]. Knockdown of *hspg2* resulted in abnormal mandibular jaw joint formation and disrupted late- stage differentiation of CNCCs, with little to no effect on early stage CNCC development. Collectively, our results suggest that *hspg2* is essential for joint formation in the developing zebrafish.

## Results

### Morpholino-induced knockdown of *hspg2* is associated with craniofacial phenotypes

It has previously been reported that *hspg2* mRNA is expressed ubiquitously throughout the head, eyes, and somites of the zebrafish. Additional immunohistochemistry staining revealed positive expression of perlecan in these regions from embryos at 2–3 h post fertilization (HPF) to 5 days post fertilization (DPF) [26, 27]. Based on this expression pattern and previous murine studies establishing that mutation of *Hspg2* results in failure of the chondro-osseous junction of developing bones and craniofacial abnormalities [13], we hypothesized that morpholino mediated knockdown of the zebrafish *hspg2* gene would cause craniofacial abnormalities.

**Fig. 1:**
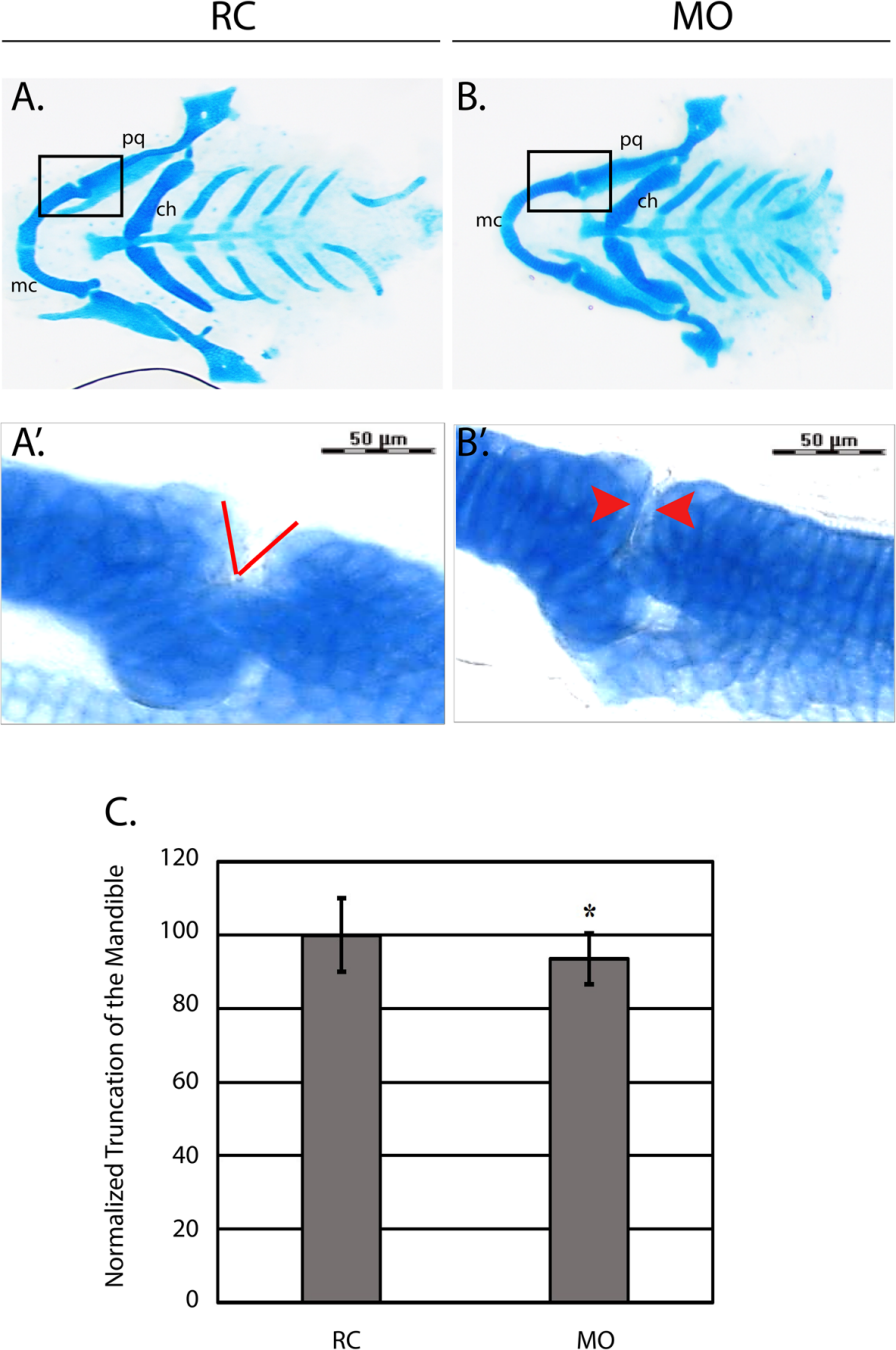
Knockdown of *hspg2* is associated with craniofacial phenotypes. **a-b** Random control (RC) and morphant (MO) groups were stained with Alcian blue at 5 days post fertilization (DPF) (*N* = 20 per group). Ceratohyal, Meckel’s cartilage, and the palatoquadrate are labeled as the abbreviations (ch), (mc), and (pq) respectively. The black box on each of the images shows the mandibular jaw joint. **a’-b′** The mandibular jaw joint enclosed by the black box at 40X magnification. a’ shows a red wedge indicating the normally developed gap in the mandibular jaw joint and b′ shows two red arrows which indicate an abnormally tight proximity between the two sides of the joint. **c** The distance between the top of the Meckel’s cartilage and top of the ceratohyal was measured across both groups (*N* = 20 per group) as a readout for micrognathia. Mandibular length was normalized to the random control (RC) group. *p = 0.025

We performed Alcian blue staining to detect craniofacial abnormalities in the developing cartilage. We measured the distance from the top of the ceratohyal to the tip of the Meckel’s cartilage as a read out for mandibular truncation as was previously described [28]. Measurements of the distance between the top of the ceratohyal and Meckel’s cartilage at 5 DPF (Fig. 1a, b, and c) showed that the injection of the *hspg2* translation blocking morpholino caused a 7% truncation in the zebrafish mandible when compared to the random control group. The observed truncation was subtle in morphants and did not appear to be the consequence of a malformed Meckel’s cartilage or defects in the development of the ceratohyal. However, upon higher magnification, *hspg2* morphants demonstrated an abnormal mandibular jaw joint between the Meckel’s cartilage and palatoquadrate (the dorsal component of the mandibular arch), a phenotype that was not present in the random control group (Fig. 1a’-b′).

### *nkx3.2* expression is decreased in *hspg2* morphants

**Fig. 2:**
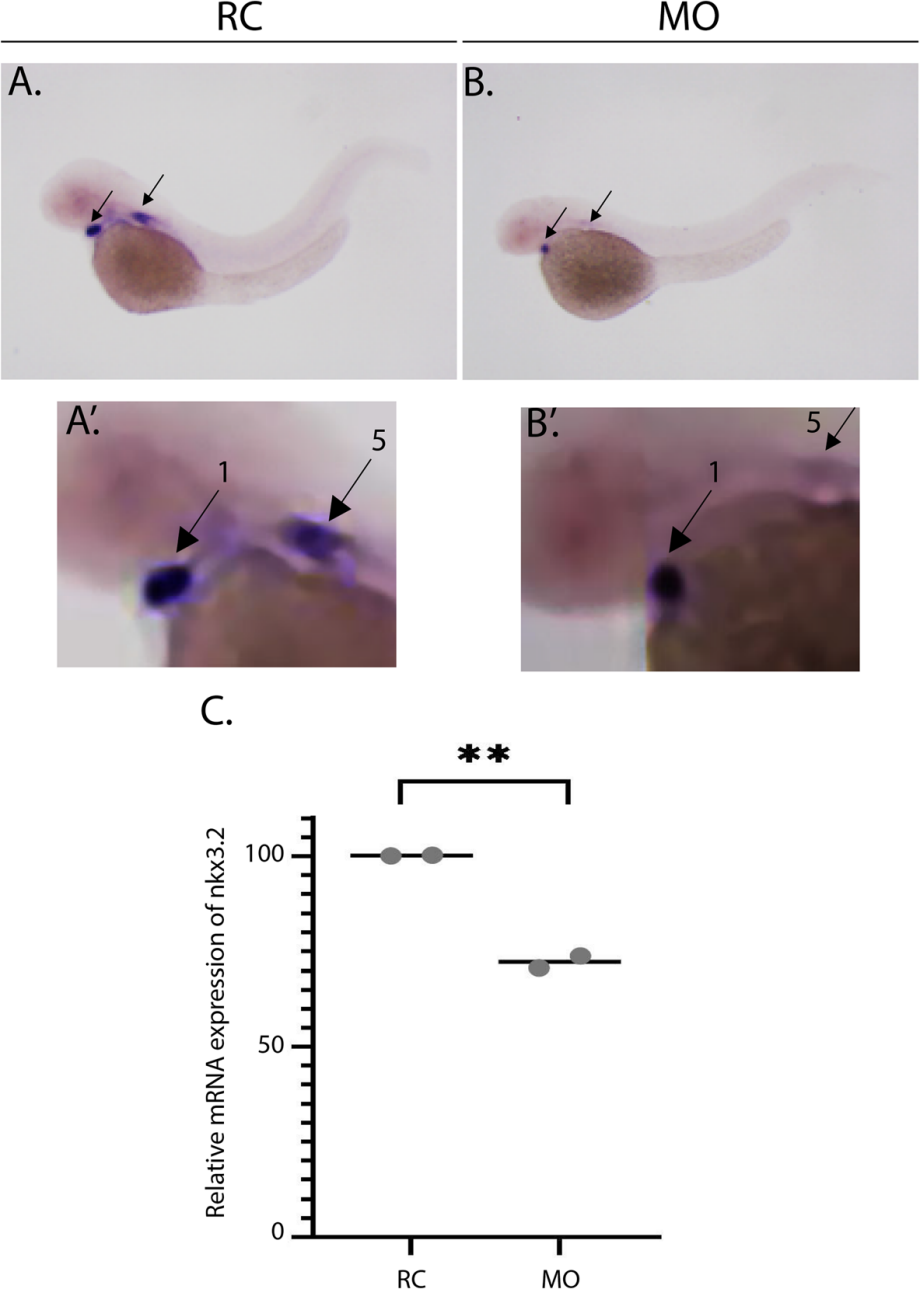
*nkx3.2* expression is decreased in *hspg2* morphants. **a-b** Whole mount in situ hybridization (ISH) was performed to detect the expression of *nkx3.2* at the 2 days post fertilization (DPF) stage. Embryos were injected as described in the methods section and subjected to ISH to detect *nkx3.2* expression in the developing jaw joint region. Black arrows indicate the expression of *nkx3.2*. There were *N* = 18 in the random control (RC) group and *N* = 14 larvae in the *hspg2* morphants (MO) group. **a’-b′** shows regions of *nkx3.2* expression. Number one and attached arrow indicate the first pharyngeal arch and number five and attached arrow indicate the fifth pharyngeal arch. **c** qPCR was performed to detect the expression of *nkx3.2* at 4 DPF on two independent occasions, each represented by a gray dot. Each biological replicate had a minimum of 7 larvae/group and a total *n* = 15 across both replicates. **p = 0.004

NK3 homeobox 2 (*nkx3.2)* was first identified in the *Drosophila melanogaster* model (where it is known as *bapx1*) and is part of the NK family of homeobox genes [29]. Homologues of the gene have been found in vertebrates and are expressed predominately in the first pharyngeal arch (a developmental structure that gives rise to the mandible), where the gene is essential for proper joint formation [29, 30]. Knockdown of *nkx3.2* causes abnormal jaw joint phenotypes (primarily fusion phenotypes) in amphibians and zebrafish [29, 31]. Therefore, we utilized *nkx3.2* expression as a marker of mandibular jaw joint development. In situ hybridization performed at 2 DPF (Fig. 2a-b) demonstrated decreased expression of *nkx3.2*. *nkx3.2* is primarily expressed in the pharyngeal arches, where we observed high expression in the 1st and 5th pharyngeal arches (Fig. 2a’-b′) (expressed posterior to the eye, annotated by the black arrows) and in the sclerotomal derivatives [31]. Knockdown of *hspg2* resulted in decreased expression of *nkx3.2* in the pharyngeal arches when compared with the random control group. Subsequent qPCR at 4 DPF confirmed a statistically significant decrease in *nkx3.2* in morphants relative to the random control group (Fig. 2c).

### Neural crest cells migrate normally in the absence of *hspg2*

**Fig. 3:**
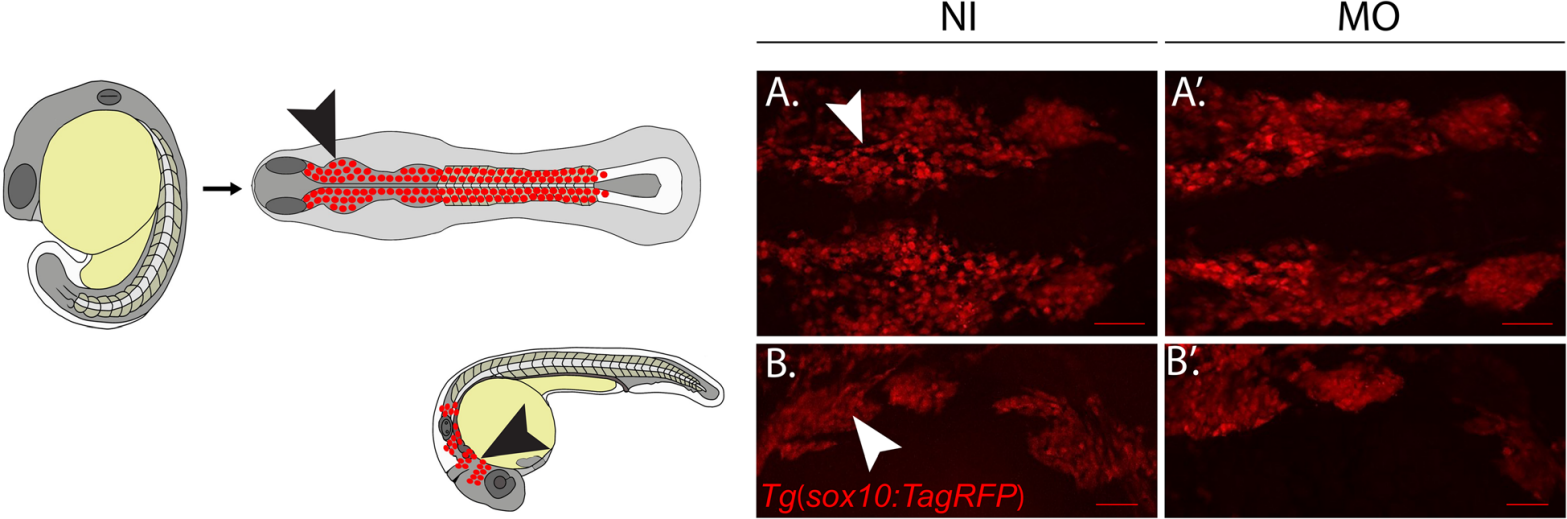
Early neural crest cell (NCC) migration and specification are normal in morphants. **a-a’** Non-injected (NI) *Tg*(*sox10:TagRFP)* larvae and *hspg2* morpholino injected *Tg*(*sox10:TagRFP)* larvae (MO) (*N* = 6 and *N* = 4, respectively) were staged and fixed at the 18-somite stage. Images are scaled at 200 μm and demonstrate two lateral streams of Sox10^+^ migrating NCCs. **b-b′** NI and MO larvae (*N* = 8 and *N* = 16 respectively) were staged and fixed at the Prim-5 stage. Images are scaled at 200 μm and demonstrate Sox10^+^ NCCs in the pharyngeal arches. No significant changes were found between the two groups at either timepoint. Schematic represents the two migratory NCC streams in a zebrafish larvae at 18 somites and the localization of the CNCCs in the four pharyngeal arches at the Prim-5 stage

Because defects in the number and migration of NCCs are possible mechanisms by which craniofacial deficits may arise [28], we hypothesized that the craniofacial abnormalities present at 5 DPF might be due to early CNCC defects. To determine if *hspg2* affects early CNCCs, we analyzed *Tg(sox10:TagRFP)* embryos at both the 18 somite (aligning with early NCC specification and migration) [32] and Prim-5 (corresponding with NCCs invading the pharyngeal arches) stages. Cells in the NCC lineage express SRY-box transcription factor 10 (*sox10)* at various stages of development where it maintains their survival, specification, and differentiation [33]. Previous studies have used *sox10* expression as a valid marker for visualizing NCCs during early developmental stages [28, 34]. Results revealed no discernable differences in the location or degree of RFP expression in *hspg2* morphants relative to control (Fig. 3a-a’ and 3b-b′).

### *dlx2a* expression in morphants is unaffected

**Fig. 4:**
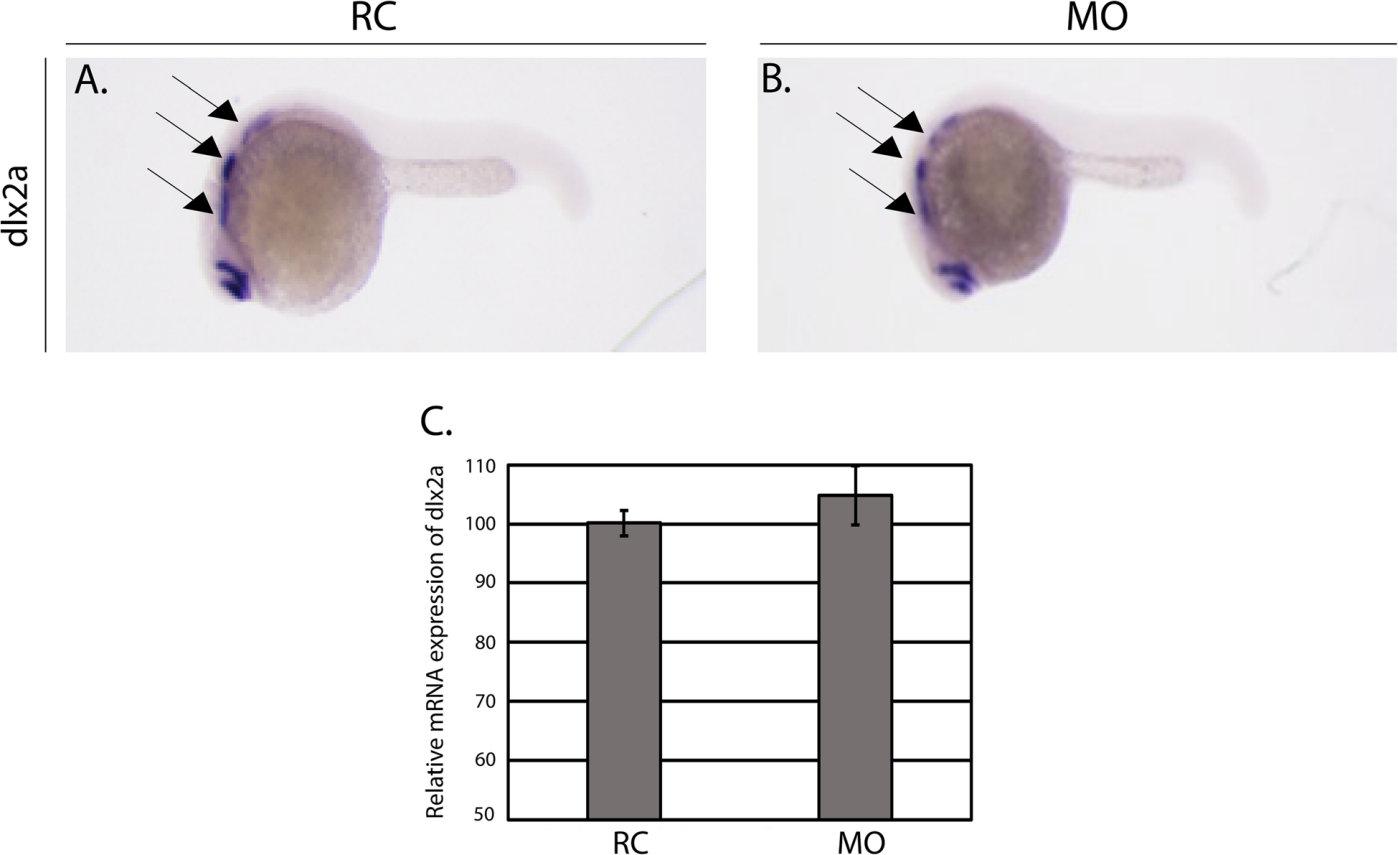
*dlx2a* expression is normal in morphants. **a-c** Whole mount in situ hybridization (ISH) was performed to detect the expression of *dlx2a* at the Prim-5 stage (*N* = 12 larvae in random control (RC) group, and *N* = 11 larvae in *hspg2* morphant (MO) group). Embryos were injected as described in the methods section and subjected to ISH to detect *dlx2a* expression in the pharyngeal arches labeled by black arrows. **d** qPCR was performed to detect the expression of *dlx2a.* Total RNA was isolated from RC and *hspg2* MO samples (*N* = 10 per group); error bars represent the standard deviation of technical replicates obtained from a pool of 10 embryos/group

We next analyzed the expression of distal-less homeobox 2a (*dlx2a)* at the Prim-5 stage in random control and *hspg2* morpholino injected embryos to determine if CNCC specification occurs normally upon knockdown of *hspg2*. This homeobox gene is expressed in CNCCs migrating to the pharyngeal arches [35] and has been established as a marker of proper CNCC specification [28, 34, 36]. In situ hybridization (Fig. 4a-c) revealed that there was no significant difference in the expression of *dlx2a* in morphants relative to random control injected embryos. qPCR measurements performed at Prim-5 in both RC and MO groups validated the normal level of *dlx2a* expression in morphant animals (Fig. 4d). Collectively, these data suggest that early CNCC development was normal.

### *hspg2* knockdown affects cell numbers in the jaw joint region

Based on our results, which suggest that the early NCC lineage was not affected, we next hypothesized that *hspg2* was mediating late stage CNCC differentiation and through this, potentially mediating the defects found at 5 DPF. To test this, we performed analysis of Sox10^+^ cells at 3 and 4 DPF using *Tg(sox10:TagRFP)* larvae. Chondrocytes across both random control and *hspg2* morphant groups at 3 and 4 DPF had normal morphology and columnar arrangement. Chondrocytes to the left of the joint were more closely clustered together than those on the right and therefore, only cells from 3 rows to the left of the joint were easily discernable as opposed to 5 rows of identifiable cells on the right of the joint.

**Fig. 5:**
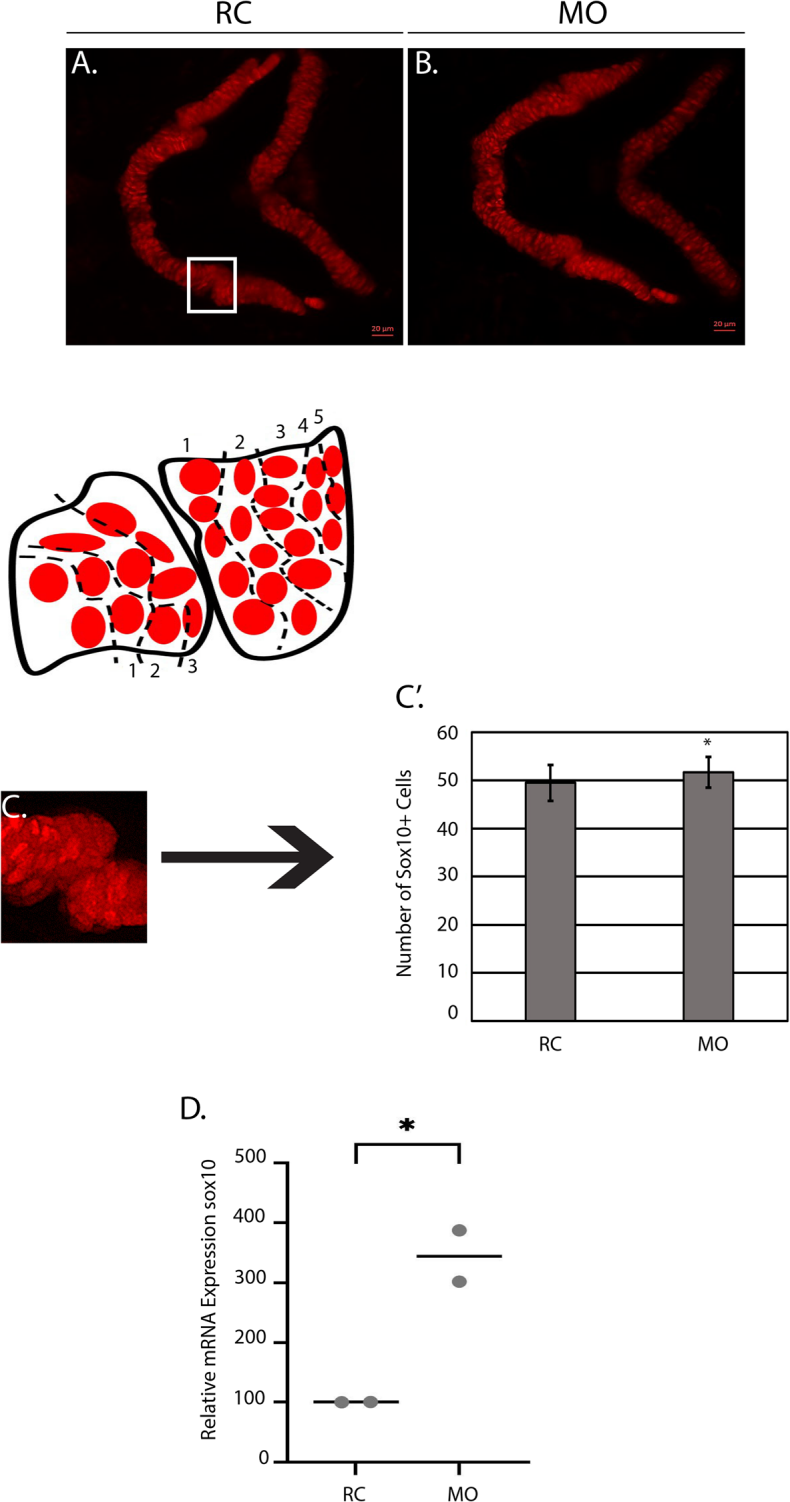
The number of Sox10^+^ cells is increased in morphants at 3 DPF. **a-b** *Tg*(*sox10:TagRFP)* random control (RC) and morphant (MO) larvae (*N* = 10 per group) were mounted in agarose and confocal images were taken at 3 days post fertilization (DPF). **c** shows the representative region where cells were quantified with a corresponding schematic showing the parameters (3 rows left, 5 rows right). **c′** Average number of Sox10^+^ cells counted across both groups (*N* = 10 per group) at 3 DPF. *P*-value pertains to the statistically significant difference between the RC group and the MOs (*p = 0.04). **d** qPCR expression of *sox10* (*N* = 24 total) in RC and MO groups at 3 DPF (***p = 0.0005). qPCR was performed on two independent occasions, each represented by a gray dot. Each biological replicate had a minimum of 12 larvae/group for a total *n* = 24

**Fig. 6:**
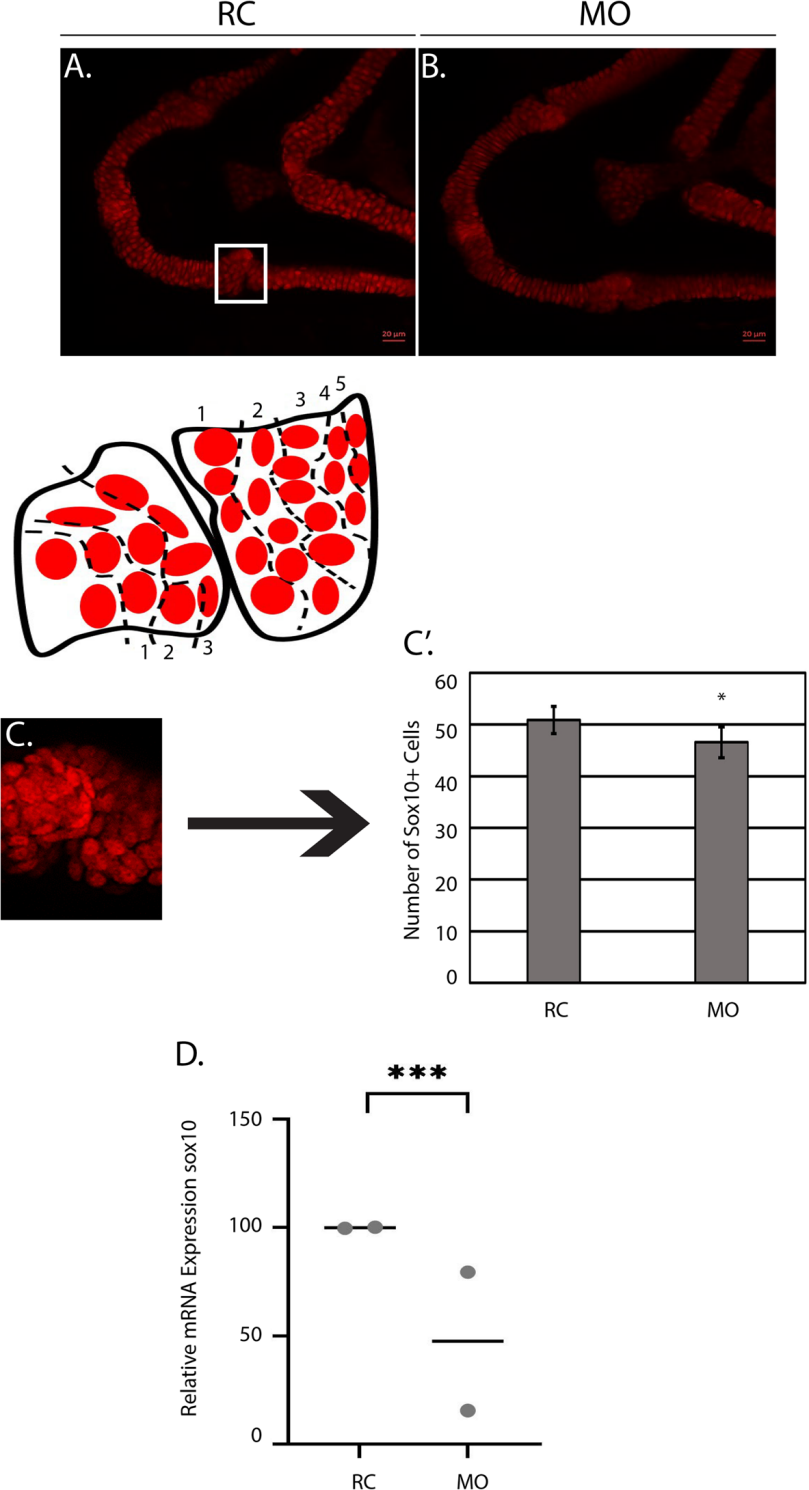
The number of Sox10^+^ cells is decreased in morphants at 4 DPF. **a-b** *Tg*(*sox10:TagRFP)* random control (RC) and morphant (MO) larvae (*N* = 10 per group) were mounted in agarose and confocal images were taken at 4 days post fertilization (DPF). **c** shows the representative region where cells were quantified with a corresponding schematic showing the parameters (3 rows left, 5 rows right). **c′** Average number of Sox10^+^ cells counted across both groups *N* = 10 per group) at 4 DPF. *P*-value pertains to the statistically significant difference between the RC group and the MO group (***p = 0.0002). **d** qPCR demonstrating the expression of *sox10* (*N* = 15 total) in RC and MO groups at 4 DPF (**p = 0.005). qPCR was performed on two independent occasions (biological replicates), each represented by a gray dot. Each biological replicate had a minimum of 7 larvae/group and a total *n* = 15

**Fig. 7:**
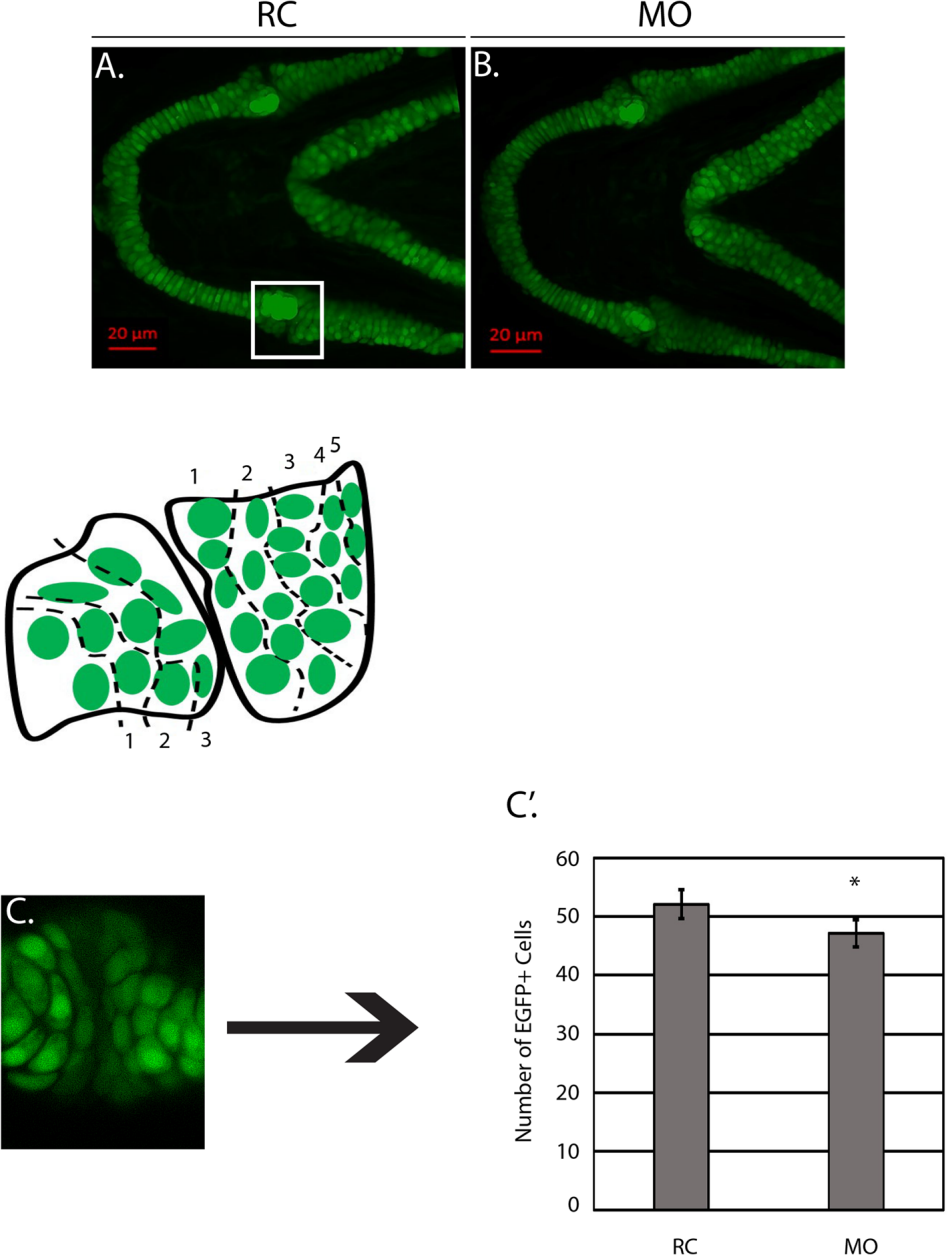
The number of Col2a1a^+^ cells is decreased at 4 DPF. **a-b** *Tg*(*col2a2a:EGFP)* random control (RC) and morphant (MO) larvae (*N* = 10 per group) were mounted in agarose and confocal images were taken at 4 days post fertilization (DPF). **c** shows the representative region where cells were quantified with a corresponding schematic showing the parameters (3 rows left, 5 rows right). **c′** Average number of Col2a1a^+^ cells counted across both groups (*N* = 10 per groups) at 4 DPF. *P*-value pertains to the statistically significant difference between the RC group and the MOs (***p = 1.44x^− 05^)

At 3 DPF (Fig. 5a-b), morphants had a statistically significant increase of Sox10^*+*^ cells in the region of interest (3 rows of chondrocytes to the left of the joint and 5 rows of chondrocytes to the right of the joint) (Fig. 5c-c’). Consistent with these results, qPCR detected an increase in *sox10* expression at 3 DPF (Fig. 5d). However, at 4 DPF (Fig. 6a-b), the number of Sox10^+^ cells were reduced relative to random control injected embryos (Fig. 6c-c’) and the level of *sox10* expression was approximately 50% of the control according to qPCR (Fig. 6d). Subsequent analysis of Col2a1a (collagen, type II, alpha 1a)^+^ cells using the *Tg (col2a1a:EGFP)* transgenic reporter in the mandibular jaw joint region revealed similar chondrocyte morphology and arrangement as samples in the *sox10* group. Cell counts (Fig. 7a-b) demonstrated a similar decline in numbers of EGFP+ cells in morphant animals at 4 DPF (Fig. 7C-C’). Collectively, these data show a progressive loss of differentiated NCCs between 3 and 4 DPF.

## Discussion

Our analysis revealed a 7% mandibular truncation and an abnormal joint phenotype in animals with knockdown of *hspg2*. Deletion of *Hspg2* in mice has been shown to cause truncated snouts, shorter and thicker mandibular structures, and flat faces [13, 37, 38], but very little has been reported on joint phenotypes and how they pertain to *hspg2* function. Although it is relatively novel, the idea of *hspg2* mediating the mandibular jaw joint region is not unfounded. Similar to other synovial joints, the mandibular jaw joint contains a synovial capsule, which, in previous cell culture work using synovial cells, has been shown to express and require perlecan for proper development [39, 40].

The mechanism by which perlecan mediates joint development is currently unknown. However, as discussed in the introduction, perlecan is a multi-domain protein with GAG side chains that interact with various growth factors like BMP, WNT, and FGF— all of which are essential for NCC development, chondrogenesis, and joint formation [38, 40]. Deficiencies or abnormalities in the level or activation of such pathways may account for the decreased number of Sox10+ or Col2a1a + cells at 4 DPF. Perlecan has also been found to bind to *Ihh* (Indian hedgehog) through its HS side chains, which in turn mediates the proliferation of chondrocytes [38]. It should be noted however, that many of these effects and pathways have predominately been implicated in the joints of the appendicular skeleton and that development of the mandibular jaw joint is slightly different.

We further demonstrate that knockdown of *hspg2* is associated with decreased numbers of Col2a1a^+^ cells at 4 DPF. These data suggest that *hspg2* has a function regulating CNCC differentiation, a finding that is supported by the number of Sox10^*+*^ cells at an equivalent time point. Our studies are supported by previous analysis in mice (*Hspg2*^−/−^) that demonstrated abnormal arrangement and proliferation of chondrocytes in the appendicular skeleton [13, 37]. It must be noted however, that although these data support one another, the cells of the appendicular skeleton derive from a different germ layer (the mesoderm) than the cells of the craniofacial skeleton (the ectoderm). Both cell lineages give rise to cartilaginous structures, but the mechanisms by which each population differentiates are likely to be different, prompting further studies. One possible future direction of our work could be to determine the interplay between perlecan and FGF because perlecan binds to FGF-2, which increases the expression of *Sox9* in vitro [39, 41]. In mice, the protein output of *Sox9* is vital to chondrogenesis because it activates *Col2a1* expression [42]. It is possible however, that there are various mechanisms underlying the function of *HSPG2* in joint development because the HS side chains of perlecan are known to bind to collagen II [12], suggesting a direct function for perlecan in chondrogenesis. Interestingly, we also observed an initial increase in the number of Sox10+ cells at 3 DPF, which at the onset seems to counter the results observed at 4 DPF. However, this increase of cells could be due to a period of proliferation in chondrocytes before maturation followed by increased cell death between 3 and 4 DPF. Further studies in this area are warranted.

Knockdown of *hspg2* was also associated with reduced expression of *nkx3.2* at 4 DPF. These results, when understood in the context of the decrease of Col2a1a^+^ cells found at 4 DPF, appear to match previous results performed in mesenchymal cell culture where *Nkx3.2* upregulates *Col2a1* by directly binding to the promoter [43]. In this situation, diminished expression of *Nkx3.2* appears to be directly proportional to a decrease in Col2a1+ cells and the differentiation of chondrocytes. It is not clear if *hspg2* directly modulates *nkx3.2* expression or if the decreased expression is simply the result of defects in the mandibular jaw joint, but studies performed in the chick have shown that *Nkx3.2* and *Sox9* cooperate to promote chondrogenic differentiation and serve as mediators of Sonic Hedgehog (Shh)-induced chondrogenesis [44]. This could be one of the mechanisms by which perlecan indirectly mediates the expression of *nkx3.2* and it would prove to be a novel discovery. Recently, it was shown that *Nkx3.2* null animals are viable, making it possible to study this gene in relation to *hspg2* without early lethality [45].

In this paper, we used morpholino-mediated knockdown of *hspg2* in zebrafish as an alternative animal model to study the role of *hspg2* during craniofacial development. Induced knockouts in the murine model have resulted in embryonic lethality from mass hemorrhaging in the pericardial cavity and severe chondrodysplasia, both occurrences which can be temporarily circumvented in the developing zebrafish [26]. To circumvent these limitations, three additional mouse models have been produced: the first model lacks exon 3, causing loss of the 3 HS side chains [7], the second is modeled after an SJS patient mutation where there is a G to an A substitution theorized to cause a misfolded protein [37, 46], and the third is a model where early lethality is restored via tissue specific expression of *Hspg2* in chondrocytes [47].

While the first two models are viable and can be used to examine adult skeletal phenotypes, the first is centered around exploring the loss of only one domain and the second is mimicking more subtle phenotypes associated with SJS. Our project seeks to understand the role of perlecan in craniofacial development using a null phenotype, a feat not easily done in a murine model. Zebrafish allow for this type of exploration because unlike mice, they are externally fertilized. This external fertilization enables the study of craniofacial development at early developmental stages, particularly with the use of transgenic fish to target specific cells [48, 49]. The third model described restores early lethality using a chondrocyte promoter and consequently cannot be utilized to study chondrogenesis or craniofacial development [47]. Zebrafish craniofacial development is conserved and the development of the viscerocranium, including the development of the pharyngeal arches, the migration and specification of NCCs, their differentiation, and signaling pathways involved have all been well characterized [24, 50]. Additionally, because the zebrafish mandibular joint is a synovial joint which develops in a similar fashion to other vertebrate synovial joints, the uncovered mechanisms of this research could be translational to other models [51].

Zebrafish are remarkably easy to manipulate genetically and have been used to great success in genetic studies. The zebrafish genome shares a high degree of genetic similarity with humans and thus provides a manner in which to understand gene function and mechanisms [25]. 70% of human genes have one zebrafish ortholog and 82% of the genes associated with morbidity also have at least one zebrafish ortholog [52]. CRISPR mutagenesis has emerged as a manner of genetic manipulation readily tolerated in the zebrafish [53] and future studies developing a germline non-sense mutant of *hspg2* are warranted.

All work reported here has been completed by use of a single translation-blocking morpholino. While translation-blocking morpholinos are a simple and effective way in which to knockdown genes of interest, they have been associated with off target effects and non-specific cell death. We recognize that utilization of a single translation-blocking morpholino is a primary limitation to our approach. The *hspg2* open reading frame is very large and difficult to perform mRNA restoration experiments with; this limited our ability to restore the morphant phenotype. In addition, we designed a splice inhibiting morpholino to the 3′ splice acceptor of exon 2, but the designed morpholino did not delete exon 3 as predicted, even at the highest concentration injected (2 nl of a 0.9 mM stock solution). We did, however, utilize a random control morpholino to account for the possibility of morpholino-induced cell death, an endeavor that proved to be rather successful in previous studies [54, 55]. And, while injection of HS side chains is a potential rescue for the morphant phenotype we observe, there is the possibility that HS/heparin co-injection would fail to rescue because a domain outside of domain I is also essential for regulation of CNCCs. Therefore, such an experiment is unlikely to demonstrate a full rescue. Collectively, these limitations reveal a potential caveat to our work in that we study a morpholino-derived phenotype. However, our data with one morpholino is supported by previous studies, including those completed using the murine model [13, 37]. In these studies, numbers of chondrocytes in the lateral skeleton are depleted, chondrocytes congregate abnormally, and mutant mice exhibit craniofacial abnormalities. Ultimately, the work herein, like many other morphant phenotypes provides a rationale for the creation of a germline mutant of *hspg2*.

Despite the limitations to our work, we demonstrate an additional function of *hspg2* in craniofacial development by examining the mandibular jaw joint region specifically. We understand that a germline mutant is required to substantiate our work, but our data is supported by previous studies, suggesting that what we observed is not a consequence of off-target effects. Nevertheless, future studies are required.

## Conclusion

In summary, our results have created novel implications for the role of *hspg2* in the development of the mandibular jaw joint, a region of the craniofacial skeleton for which development has not been well elucidated. Furthermore, connections between *hspg2* and *nkx3.2* have yet to be drawn in literature. Additional data concerning late CNCC differentiation raises pertinent questions about the fate of certain cells within different areas of the developing face. Although future studies to more cohesively understand the role of *hspg2* in craniofacial development are needed, these data lay significant groundwork for future experiments in this field and suggest that zebrafish are an acceptable model to study the function of *hspg2* in craniofacial development.

## Methods

### Animal care

For all experiments, embryos were obtained by crossing adult *Tg(sox10:tagRFP*), *Tg*(*col2a1a*:*EGFP*), or AB wildtype fish. Embryos were maintained in E3 embryo medium at 28 °C. All zebrafish were maintained at The University of Texas El Paso according to the Institutional Animal Care and Use Committee (IACUC) guidelines protocol 811,689–5. All adult fish were obtained from the University of Colorado, Anschutz Medical Campus or the Zebrafish International Resource Center (ZIRC). Adult and larval zebrafish were euthanized and anesthetized according to guidelines from the 2020 American Veterinary Medical Association (AVMA) which indicate 30 min at RT in 10 g/L buffered solution of MS222 in conjunction with a cold ice bath for adults. AVMA guidelines for embryos younger than 7 DPF were followed, which indicate 1–10% sodium hypochlorite solution following anesthesia. Detailed methods for anesthesia and euthanasia have been previously described [54].

### Antisense oligonucleotide morpholino design and microinjection

Two antisense oligonucleotide morpholino sequences were designed in conjugation with Gene Tool LLC. The first was a translation blocking morpholino (MO) with the sequence 5′-TATCCTCGCCCCCATTTCTGCCAA-3′, created to bind to the *hspg2* translation start site and sterically knockdown perlecan translation in the developing larvae. The second was a random control morpholino with the sequence 5′-AAAAAAAAAAAAAAAAAAAAAAAA-3′. This random control morpholino was used to assure that the translation blocking MO microinjections were not causing any form of extraneous cell death as previously described [56].

The initial concentration gradient for the translation blocking morpholino was as follows: 0.10 mM (1.65 ng/embryo), 0.30 mM (4.95 ng/embryo), 0.50 mM (8.25 ng/embryo), and 0.70 mM (11.55 ng/embryo). A high mortality rate not attributed to lack of fertilization was found in larvae injected at higher concentrations (0.50 mM and 0.70 mM) when compared to wildtype group at 1 DPF. Morphant larvae at 0.30 mM exhibited growth delays when examined at 1 DPF (verified through somite counts), prohibiting accurate comparison between morphant and control group. Microinjections were then performed at 1.65 ng per embryo at a volume of 0.52 nL per embryo, a concentration and volume where there was minimal mortality and no growth delay. To ensure the final phenotype was not a consequence of global deformities at later stages, larvae were measured for length using Zeiss software and staged according to length at 3 and 4 DPF.

For all experiments, the morphant experimental group is compared to either a random control group or wildtype non-injected. The randomized control morpholino has been shown through previous literature to have no associated phenotypes, indicating that it does not influence final results and therefore is the appropriate control group for comparison [54, 55, 57]. Thus, for statistical analysis, comparisons were performed using a T-test between the random control group and the morpholino. MOs were injected into embryos at the one cell stage with a stock concentration of 0.10 mM (1.65 ng/embryo) and at a volume of 0.52 nL per embryo as explained above. Wildtype larvae were non-injected and used predominately as a baseline for fertilization rates.

### Alcian blue staining and imaging

Zebrafish larvae (aged 5 days post fertilization (DPF)) were fixed in 2% PFA in PBS, pH 7.5 for 1 h at room temperature (RT). Samples were washed for 10 min with 100 mM Tris pH 7.5/10 mM MgCl2, stained with Alcian blue stain (pH 7.5: 0.4% Alcian blue (Anatech Ltd., MI) in 70% EtOH, Tris pH 7.5 (Fisher, MA), and 1 M MgCl2 (Fisher, MA)), and incubated overnight at RT. Samples were subsequently destained and rehydrated using an EtOH: Tris pH 7.5 gradient as previously described [34]. Embryos were bleached (30% H2O2 (Sigma, St. Louis, MO), 20% KOH (Fisher, MA)) for 10 min at RT. Samples were washed twice for 10 min per wash in wash buffer (25% glycerol/0.1% KOH (Fisher, MA)) and stored at 4 °C in storage buffer (50% glycerol/0.1% KOH (Fisher, MA)) until imaged. The distance between the top of the Meckel’s cartilage (the ventral component of the mandibular arch) and the top of the ceratohyal (a pharyngeal arch cartilage) was measured for each embryo as a method to analyze truncation of the Meckel’s cartilage, which would be analogous to micrognathia as described previously [28]. The final measurement is the distance between the two structures and not the full length of the Meckel’s cartilage. Distance measurements were performed using Zeiss software and all distances are in uM. For imaging, a representative sample of the two groups (*hspg2* morphants and random control larvae) were dissected and viscerocranium was mounted on a glass slide with 100% glycerol. A Leica microscope was used to take high-resolution color images of each sample.

### Whole mount in situ hybridization (ISH)

ISH was performed as previously described [54, 58, 59]. Briefly, larvae were fixed in 4% paraformaldehyde (Electron Microscopy Sciences, PA) and permeabilized with appropriate concentrations of proteinase K as recommended by [59]. Prehybridization and buffer concentrations were adopted as described previously [58]. Larvae were then incubated overnight with probe (*dlx2a* and *nkx3.2* at 127 ng) at 70 °C and developed using anti-DIG Fab fragment (Sigma) antibodies with AP substrate (Sigma). All images were captured as previously described [54, 58]. Statistical analysis was performed using a Fisher’s exact test.

### Quantitative real time polymerase chain reaction (qPCR)

qPCR was performed as previously described [54, 58] using total RNA extracted with Trizol reagent (Fisher) from a pool of larvae at each indicated time point. cDNA was produced with the Verso cDNA synthesis kit (Fisher). Sybr green (Fisher) based primer pairs for each gene analyzed are as follows: *dlx2a* fwd CCTCACGCAAACACAGGTTA, *dlx2a* rev TGTTCATTCTCTGGCTGTGC, *nkx3.2* fwd GCAGATTTAGCGGACGAGAC, *nkx3.2* rev GCTTCAACCACCAGCGTTAT, *sox10* fwd ACGCTACAGGTCAGAGTCAC, *sox10* rev ATGTTGGCCATCACGTCATG, *rpl13a* fwd TCCCAGCTGCTCTCAAGATT, and *rpl13a* rev TTCTTGGAATAGCGCAGCTT. Data presented was derived by the 2^ΔΔct^ relative fold change methodology as described [34, 54, 58, 60]. For each biological replicate, RNA was isolated from a pool of injected embryos. Morphant gene expression changes are demonstrated relative to the expression from a pooled group of individuals injected with random control morpholinos. Each qPCR analysis was performed in biological duplicate, which can be defined as two independent occasions with independent parents and an independent injection, except for the expression of *dlx2a*, which was performed using a single pool of *n* = 10 larvae and was performed to validate in situ hybridization. For each biological replicate, the qPCR was performed in technical triplicates. A single gray dot on the graphs in Figs. 2, 5, and 6 demonstrates the average of the three technical replicates with each gray dot representing a unique biological replicate. Statistical analysis of messenger RNA (mRNA) expression was performed using a standard t-test on biological replicates, by taking the average and standard deviation of each biological replicate.

### Confocal imaging and transgenic cell counts

Transgenic larvae (*Tg(sox10:tagRFP*) and *Tg(col2a1a:EGFP*)) were fixed at the stated time points using 4% paraformaldehyde. Fixed larvae were mounted in 0.6% low-melt agar in a glass bottom dish (Fisher). Imaging was performed on a Zeiss LSM 700 at 20X and 40X Oil magnification. Images were restricted to the larval craniofacial region. For each fish, a minimum of 20 to 30 z-stacks were collected. At 3 DPF, micron depth across the *Tg(sox10:TagRFP)* random control group ranged from 60 to 100 μm while micron depth ranged from 57 to 84 μm in morphants. Micron depth across the 4 DPF *Tg*(*sox10:TagRFP)* random control group ranged from 63 to 100 μm and from 66 to 93 μm in the morphant group. At 4 DPF *Tg(col2a1a:EGFP)* random control group micron depth ranged from 63 to 99 μm and from 72 to 90 μm in the morphant group.

The number of cells per z-stack (20–30 stacks/fish) at both jaw joints were manually counted using the ImageJ cell counter, which marks each individual cell and keeps track of total cell numbers. Region of interest around the jaw joints were selected to be 3 rows of chondrocytes on the left side of the joint and 5 rows on the right. This region was selected because these cells were consistently visible across z-stacks. Statistical significance was obtained by using a Student t-test with random control and morphant data [58].

## Data Availability

All data analyzed during this study are included within this published article and any supplementary material. Raw data is available upon request from the corresponding author.

## Abbreviations

*HSPG2/Hspg2/hspg2*: Heparan sulfate proteoglycan 2
dpf: Days-post-fertilization
NCC: Neural crest cells
*sox10*: SRY-box transcription factor 10
*nkx3.*2: NK3 homeobox 2
SJS: Schwartz-Jampel Syndrome
DDSH: Dyssegmental Dysplasia Handmaker Type;
qPCR: Quantitative polymerase chain reaction
*col2a1a*: Collagen, type II, alpha 1a
MO: *Hspg2* morphants
NI: Non-injected wildtype
RC: Random control group
